# Time-related changes in hepatic and colonic mitochondrial oxygen consumption after abdominal infection in rats

**DOI:** 10.1186/s40635-018-0219-9

**Published:** 2019-01-08

**Authors:** Anna Herminghaus, Henrike Papenbrock, Rebecca Eberhardt, Christian Vollmer, Richard Truse, Jan Schulz, Inge Bauer, Adelheid Weidinger, Andrey V. Kozlov, Johnny Stiban, Olaf Picker

**Affiliations:** 1 Department of Anaesthesiology, University of Düsseldorf, Moorenstrasse 5, 40225 Düsseldorf, Germany; 2 Ludwig Boltzmann Institute for Clinical and Experimental Traumatology, AUVA Research Center, Donaueschingenstraße 13, 1200 Wien, Austria; 3 Department of Biology and Biochemistry, Birzeit University, Birzeit, Ramallah, Palestine

**Keywords:** Abdominal infection, Sepsis, Time course, Mitochondrial function, Liver, Colon

## Abstract

**Background:**

Evidence suggests that early adaptive responses of hepatic mitochondria occur in experimentally induced sepsis. Little is known about both colonic mitochondrial function during abdominal infection and long-term changes in mitochondrial function under inflammatory conditions. We hypothesize that hepatic and colonic mitochondrial oxygen consumption changes time-dependently after sterile laparotomy and in the course of abdominal infection. The aim of the present study was to investigate the hepatic and colonic mitochondrial respiration after sterile laparotomy and abdominal infection over up to 96 h.

**Methods:**

After approval of the local Animal Care and Use Committee, 95 Wistar rats were randomized into 8 groups (*n* = 11–12): 1–4 sham (laparotomy only) and 5–8 colon ascendens stent peritonitis (CASP). Healthy, unoperated animals served as controls (*n* = 9). The mitochondrial respiration in colon and liver homogenates was assessed 24, 48, 72, and 96 h after surgery. Mitochondrial oxygen consumption was determined using a Clark-type electrode. State 2 (oxygen consumption in the presence of the substrates for complexes I and II) and state 3 respiration (ADP dependent) were assessed. The respiratory control ratio (RCR state 3/state 2) and ADP/O ratio (ADP added/oxygen consumed) were calculated for both complexes. Data are presented as means ± SD, two-way ANOVA followed by Tukey’s post hoc test.

**Results:**

Hepatic RCR was initially (after 24 h) elevated in both operated groups; after 48 h only, the septic group was elevated compared to controls. In CASP groups, the hepatic ADP/O ratio for complex I was elevated after 24 h (vs. controls) and after 48 h (vs. sham) but declined after 72 h (vs. controls). The ADP/O ratio for complex II stayed unchanged over the time period until 96 h.

The colonic RCR and ADP/O did not change over time after sham or CASP operation.

**Conclusion:**

Hepatic, but not colonic, mitochondrial respiration is increased in the initial phase (until 48 h) and normalizes in the longer course of time (until 96 h) of abdominal infection.

**Electronic supplementary material:**

The online version of this article (10.1186/s40635-018-0219-9) contains supplementary material, which is available to authorized users.

## Background

The mortality rate in sepsis, septic shock, and the consecutive multiorgan dysfunction syndrome (MODS) in humans remains very high (30–40%); various attempts to modulate the inflammatory response and prevent organ damage failed to improve survival [1, 2]. Intraabdominal infections are the second most common cause for sepsis and thus represent an important clinical problem [3]. Despite the high incidence of diseases such as systemic inflammatory response syndrome (SIRS), sepsis, and MODS, their pathophysiology is not clearly understood [4]. Microcirculatory and mitochondrial dysfunction are considered to be some of the possible pathophysiological mechanisms in septic shock and MODS [5, 6]. Nevertheless, the role of mitochondria in the manifestation of sepsis pathophysiology remains controversial. It is still debatable whether mitochondria are the initiators, amplifiers, targets, or only bystanders in sepsis-induced organ dysfunction [7]. On the one hand, dysoxia and oxidative stress can cause mitochondrial dysfunction, and on the other hand, mitochondria can produce reactive oxygen species (ROS) which facilitate oxidative stress reactions [7–9].The mitochondrial respiratory response during sepsis is tissue-specific and varies remarkably between organs [10, 11]. Multiple studies on hepatic mitochondrial function in sepsis failed to reach a consensus and are therefore controversial [12]. Following septic injury, studies have demonstrated impaired [13–15], unaffected [16, 17, 21], or even improved mitochondrial function [18–20]. Whereas studies on hepatic mitochondria are numerous, studies on mitochondrial function of other organs are lacking.

A notable exception is the study performed by Mittal and colleagues which showed decreased mitochondrial function in the jejunum but not in the duodenum 6 h after induction of acute pancreatitis [21]. King et al. could also show decreased ileal mucosal oxygen consumption for samples derived from endotoxemic rats compared to controls [22]. Nevertheless, data about mitochondrial function in other parts of the gut, e.g. the colon during septic conditions, are not available even though the colon plays a very important role during sepsis. Even if the colon is not the primary focus of infection, inflammation could be worsened when the barrier function is damaged [23]. We have previously shown that hepatic mitochondrial function depends on the severity of sepsis [18]. Operative stress induced by laparotomy and, to a greater extent, moderate sepsis led to a higher energy coupling within 24 h without affecting the efficacy of oxidative phosphorylation, whereas more severe sepsis did not affect hepatic mitochondrial function. This suggests an adaptive response in the early phase of inflammatory reaction. However, there are no data on mitochondrial function at other time points during the course of sepsis and healing of sterile laparotomies. Evidence suggests that mitochondrial function changes during the inflammatory response. It has been shown that mitochondrial respiration improves in the early phase of acute critical illness such as sepsis and deteriorates during the late phase [24]. To date, there have only been a few studies describing time-dependent mitochondrial function during sepsis. Brealey et al. have examined mitochondrial function in rat liver and skeletal muscle over the course of 72 h of sepsis induced by faecal peritonitis and could show that the activities of the individual respiratory chain complexes changed differentially in this time period. Moreover, they could show a stronger correlation of changes of mitochondrial function with the severity of sepsis than with the course of time [14].

We hypothesize that hepatic and colonic mitochondrial oxygen consumption changes time-dependently after sterile laparotomy and in the course of abdominal infection. To test this, we analysed mitochondrial function of the liver and the colon in over the course of abdominal infection for up to 96 h in a rat model.

## Materials and methods

### Animals

The study was approved by the local Animal Care and Use Committee (Landesamt für Natur, Umwelt und Verbraucherschutz, Recklinghausen, Germany), and all experiments were performed in accordance with the NIH guidelines for animal care.

A total of 95 adult male Wistar rats (374 ± 23 g body weight) were kept at an artificial 12-h light/dark cycle at constant room temperature and humidity with free access to standard chow and tap water. They were randomized into 8 groups: groups 1–4 (*n* = 12), sham-operated animals (laparotomy with the stent fixed on the outside wall of the gut), and groups 5–8 (*n* = 11–12), colon ascendens stent peritonitis (CASP with a 14-G stent). The control group consisted of 9 healthy untreated animals.

### CASP/sham operation

Polymicrobial abdominal infection was induced by the implantation of a 14-G stent into the wall of the large intestine to promote faecal leakage into the abdominal cavity (colon ascendens stent peritonitis surgery (CASP)) as previously reported [18, 25].

Briefly, anaesthesia was induced and maintained by the volatile anaesthetic sevoflurane (3.0 Vol%, FiO_2_ 0.5). The opioid buprenorphine was applied at 0.05 mg/kg s.c. An approximately 2-cm median incision of the abdomen was performed, the gut was located, and an 8-mm-long 14-G stent was fixed, penetrating the colonic wall (ca. 0.5 cm distal to the caecum). Cautious palpation of the gut and gentle milking of faeces into the stent ensured constant leakage into the abdomen. Sham animals were anaesthetized and laparotomized as stated above except that the stent was fixed on the outside wall of the gut without penetrating it. The gut was carefully returned to the abdominal cavity, and the abdominal wall was closed. The animals were placed in a warmed cage to recover from anaesthesia. At 24, 48, 72, or 96 h after CASP or sham operation, animals were euthanized with pentobarbital i.p. (120 mg/kg). Blood was obtained by cardiac puncture. A laparotomy was performed, and the right lobe of the liver and colon tissue (1 cm distal from the stent) were harvested and immediately placed into 4 °C-cold isolation buffer (200 mM mannitol, 50 mM sucrose, 5 mM KH_2_PO_4_, 5 mM morpholinepropanesulfonic acid (MOPS), 0.1% fatty acid-free bovine serum albumin (BSA), 1 mM ethylenediaminetetraacetic acid (EDTA), pH 7.15).

As described previously [18, 25], we used a numeric score sheet (Clinical Score—see Table 1) to estimate the severity of sepsis (loss of body weight, appearance, spontaneous behaviour, provoked behaviour, breathing frequency, expiratory breathing sound, abdominal palpation, and condition of droppings). The animals were examined every 12 h. Animals scoring more than 10 points were euthanized. A single, non-blinded investigator performed the scoring. In the defined time frame (24, 48, 72, and 96 h after operation), animals received analgesia (buprenorphine 0.05 mg/kg in 0.6 ml NaCl s.c every 12 h). No antibiotics and no additional fluid therapy were applied.

**Table 1:** Clinical score (all changes refer to the baseline)

Examination	Results	
Body weight	Weight loss in %	% < 5 ⇒ 0 P % 5–15 ⇒ 2 P % 15–20 ⇒ 3 P % > 20 ⇒ 10 P
Appearance	1. Normal appearance, fur smooth, clean	⇒ 0 P
	2. Slight grooming deficiency, rough fur	⇒ 1 P
	3. Increasing grooming deficiency, rings around eyes, anus	⇒ 2 P
	4. Clear grooming deficiency, crusty eyes, bedding sticks to anus	⇒ 3 P
Spontaneous behaviour	1. Rat investigates cage, active	⇒ 0 P
	2. Rat remains in one place, movement of entire body present	⇒ 1 P
	3. Hunched posture, swaying gait	⇒ 3 P
	4. Immobile, lateral position	⇒ 10 P
Provoked behaviour	1. Rat flees when opening cage, strong muscle tonus	⇒ 0 P
	2. Rat flees when hand approaches	⇒ 1 P
	3. Rat flees when touched	⇒ 2 P
	4. No flight reaction	⇒ 3 P
Breathing frequency	Difference in %	% < 10 ⇒ 0 P % 10–20 ⇒ 1 P % 20–50 ⇒ 2 P % > 50 ⇒ 3 P
Expiratory breathing sound	No	⇒ 0 P
	Yes	⇒ 1 P
Abdominal palpation	1. No pain when applying pressure, soft abdomen	⇒ 0 P
	2. Slight reaction to abdominal palpation, soft abdomen	⇒ 1 P
	3. Clear pain reaction to abdominal palpation, abdominal resistance	⇒ 2 P
	4. Clear pain reaction to abdominal palpation, hard abdomen	⇒ 3 P
Condition of droppings	1. A lot of normal droppings in cage, defecating during examination	⇒ 0 P
	2. A lot of droppings in cage, droppings with blood, runny or mucous	⇒ 1 P
	3. Few droppings in cage, independent of the condition	⇒ 2 P
	4. No droppings in cage	⇒ 3 P

### Control group

Healthy, unoperated controls (*n* = 9) were euthanized with pentobarbital i.p. (120 mg/kg). Blood was obtained by cardiac puncture. A laparotomy was performed, and the right lobe of the liver and colon tissue (1.5 cm distal from the caecum) were harvested and immediately placed into 4 °C-cold isolation buffer as described above. This group serves as a control group for all investigated time points.

### Preparation of liver and colon homogenates

Liver tissue was placed in 4 °C-cold isolation buffer, minced into 2–3-mm^3^ pieces, rinsed twice in isolation buffer to remove traces of blood, and homogenized (Potter-Elvehjem, 5 strokes, 2000 rpm).

Freshly harvested colon tissue was placed in 4 °C-cold isolation buffer, quickly opened, and dried softly with a cotton compress to remove remains of faeces and mucus. After treatment with trypsin for 5 min on ice, the tissue was placed in 4 °C isolation buffer containing 20 mg/ml BSA and protease inhibitors (cOmplete™ Protease Inhibitor Cocktail, Roche Life Science, Mannheim, Germany), minced into 2–3-mm^3^ pieces, and homogenized (Potter-Elvehjem, 5 strokes, 2000 rpm).

Protein concentration in the tissue homogenates was determined by the Lowry method [26] with bovine serum albumin as a standard.

### Measurement of mitochondrial respiratory rates

Mitochondrial oxygen consumption was measured at 30 °C using a Clark-type electrode (model 782, Strathkelvin instruments, Glasgow, Scotland). Tissue homogenates were suspended in respiration medium (130 mM KCl, 5 mM K_2_HPO_4_, 20 mM MOPS, 2.5 mM EGTA, 1 μM Na_4_P_2_O_7_, 0.1% BSA for liver, and 2% BSA for colon, pH 7.15) to yield a protein concentration of 4 mg/ml or 6 mg/ml for liver and colon, respectively.

Mitochondrial state 2 respiration was recorded in the presence of either complex I substrates glutamate and malate (both 2.5 mM, G-M) or the complex II substrate succinate (10 mM for liver, 5 mM for colon, S).

The maximal mitochondrial respiration in state 3 was measured after the addition of ADP (250 μM for liver, 125 μM for colon). The respiratory control ratio (RCR) was calculated (state 3/state 2) to define the coupling between the electron transport system and oxidative phosphorylation. To reflect the efficacy of oxidative phosphorylation, the ADP/O ratio was calculated from the amount of ADP added and oxygen consumption. The average oxygen consumption was calculated as the mean from three technical replicates.

The solubility of oxygen was assumed to be 223 μmol O_2_ l^−1^ at 30 °C according to the Strathkelvin instruments manual. Respiration rates were expressed as nanomoles per minute per milligramme protein. No correction of the natural drift of the electrode was made since a drift of less than 0.5% over 12 h is neglectable in our experimental setup.

Mitochondria were checked for leakage by the addition of 2.5 μM cytochrome c and 0.05 μg/ml oligomycin. There was no increase in flux after the addition of cytochrome c, indicating integrity of the mitochondrial outer membrane. When ATP synthesis was inhibited by oligomycin, the mitochondria were transferred to the state 2, which reflects the respiration rate compensating the proton leak. The lack of difference in O_2_ consumption after adding of oligomycin compared to state 2 indicates that the inner membrane was intact and mitochondria were not damaged through the preparation procedure.

### Determination of MDA in the liver and colon

In order to measure malondialdehyde (MDA) levels, condensation of MDA with thiobarbituric acid was performed, as we reported previously [18]. Briefly, frozen liver and colon tissue (approx. 50 mg) were homogenized in 500 μl 1.5% KCl. The homogenate (250 μl) was then mixed with 1500 μl of 1% phosphoric acid and 500 μl of 0.6% 2-thiobarbituric acid (TBA) and heated to 95 °C for 45 min. After cooling, 2000 μl of butanol was added, and the obtained mixture was vortexed and then centrifuged at 2900 rcf for 15 min. The supernatant was collected, and the absorbance was spectrophotometrically measured at 535 nm and 520 nm. The malondialdehyde concentration was calculated using a MDA standard and normalized to protein concentration (determined by the Lowry method [26]) and expressed as nanomole MDA per milligramme protein.

### Plasma analysis

Plasma was obtained by centrifugation (4 °C, 4000 rcf, 10 min) of blood samples collected in EDTA tubes and stored at − 80 °C. Activities of alanine aminotransferase (ALT), aspartate aminotransferase (AST), and lactate dehydrogenase (LDH) as well as concentrations of creatinine and urea were measured in the Central Institute of Clinical Chemistry and Laboratory Medicine of the University Hospital Duesseldorf, Germany. Levels of interleukin 10 (IL-10), interleukin 6 (IL-6), and tumour necrosis factor-α (TNF-α) were measured spectrophotometrically using specific rat ELISA sets (BD Opt EIA ELISA Set, BD Biosciences, Mississauga, Canada) according to the manufacturer’s instructions.

### Statistics

GraphPad Prism v6.01 (GraphPad Software, Inc., USA) was used for statistical analysis. Data are shown as mean ± SD and two-way ANOVA was performed and followed by post hoc Tukey’s multiple comparison test. *p* < 0.05 was considered significant.

## Results

### Time-dependent effects of sham and CASP surgery on severity of abdominal infection

**Fig. 1:**
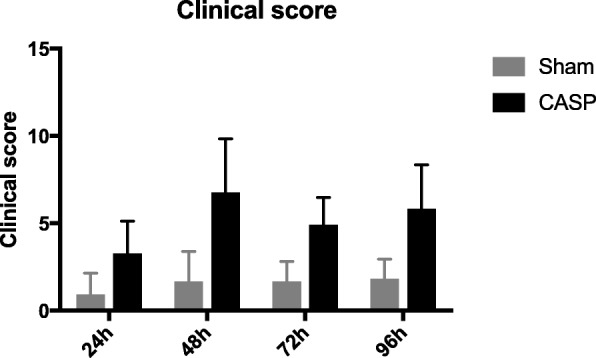
Time course of clinical score in sham- (black bars) or CASP- (grey bars) operated animals. Clinical score was monitored and assessed according to Table 1 (see the “Materials and methods” section). Data are shown as mean ± SD (*n* = 12, CASP 24 h *n* = 11)

In sham animals, there was no difference in clinical score between the investigated time points. In CASP animals, the clinical score was higher at 48 h and 96 h compared to the 24 h time point. At all time points, the clinical score was higher in the CASP groups compared to the respective sham animals (Fig. 1).

The mortality rate in our model was very low overall. Three out of 48 CASP animals died at 12, 36, and 84 h after CASP operation. No mortality was observed in the sham group.

### Effect of sterile laparotomy and abdominal infection on plasma markers of inflammation and tissue damage

**Fig. 2:**
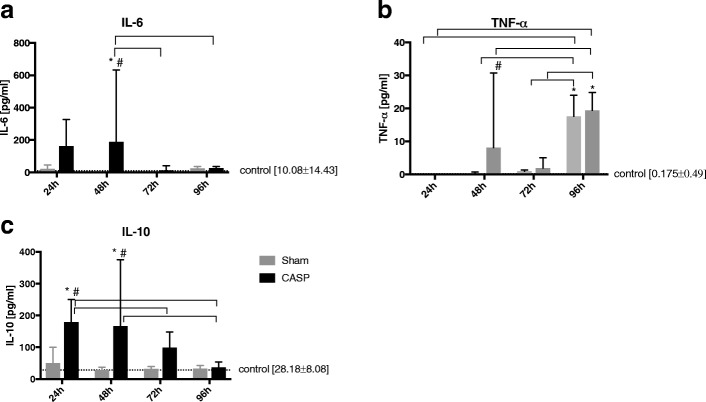
Markers of inflammation change in a time-dependent manner in the course of abdominal infection. Plasma levels of IL-6 (**a**), TNF-α (**b**), and IL-10 (**c**) were analysed at 24, 48, 72, and 96 h post operation (sham operation, light grey bars; CASP, dark grey bars). Healthy, non-operated rats served as controls (straight horizontal dotted line). Data are shown as mean ± SD (controls *n* = 9, sham *n* = 12, CASP 24 h *n* = 11, other CASP groups *n* = 12, *p < 0.05 vs. controls, #*p* < 0.05 vs. sham,  between groups

The levels of pro- and anti-inflammatory markers were analysed in the plasma of control, sham-, and CASP-operated rats. Plasma levels of the proinflammatory cytokine interleukin 6 (IL-6) increased time-dependently with a maximum at 48 h after CASP operation (compared to healthy controls and to sham animals) and thereafter dropped to physiological levels. In sham-operated animals, IL-6 levels remained unchanged over time (Fig. 2a). Tumour necrosis factor alpha (TNF-α) was significantly higher in CASP animals 48 h (compared to sham group) and 96 h (compared to healthy controls) after CASP operation. In the sham group, TNF-α showed only a late peak 96 h after surgery (Fig. 2b). Levels of the anti-inflammatory cytokine interleukin 10 (IL-10) were significantly elevated at 24 and 48 h after the CASP operation compared to healthy controls and to sham animals. At 72 and 96 h, IL-10 levels were similar to the controls. In sham-operated animals, IL-10 levels remained unchanged over time (Fig. 2c).

As for assessing kidney tissue damage, the levels of creatinine as a marker of renal function remained within the physiological range in all groups at the investigated time points (Table 2). Urea was elevated in the CASP group 48 h after CASP surgery compared to the sham and to the control group but still remained within the normal range (Table 2).

**Table 2:** Laboratory chemical parameters for organ damage: creatinine and urea for kidney; AST, ALT, and LDH for liver

	Creatinine (mg/dl)	Urea (mg/dl)	AST (U/l)	ALT (U/l)	LDH (U/l)
Control	0.32 ± 0.15	32.33 ± 5.79	67.17 ± 14.32	47.67 ± 8.87	236.83 ± 143.83
Sham 24 h	0.28 ± 0.06	39.25 ± 9.20	148.25 ± 61.92*	53.92 ± 16.53	216.50 ± 82.32
CASP 24 h	0.29 ± 0.03	33.60 ± 4.33	138.30 ± 37.48*	41.00 ± 12.21^#^	182.70 ± 24.47
Sham 48 h	0.28 ± 0.05	36.33 ± 6.34	103.33 ± 41.41	52.33 ± 7.01	211.33 ± 194.04
CASP 48 h	0.35 ± 0.18	52.16 ± 36.37^* #^	124.83 ± 52.93*	49.17 ± 16.61	237.17 ± 150.08
Sham 72 h	0.29 ± 0.03	34.75 ± 4.33	75.33 ± 11.02	49.00 ± 9.67	151.92 ± 44.8
CASP 72 h	0.31 ± 0.03	39.92 ± 4.40	65.00 ± 9.87	40.17 ± 7.92	137.67 ± 30.12
Sham 96 h	0.33 ± 0.05	37.25 ± 3.22	87.83 ± 36.12	53.67 ± 16.79	163.58 ± 75.78
CASP 96 h	0.30 ± 0.00	33.67 ± 6.20	87.83 ± 42.65	42.75 ± 9.96	180.58 ± 156.22

Data are shown as mean ± SD, *p < 0.05 vs. controls, #*p* < 0.05 vs. sham (controls *n* = 9, sham *n* = 12, CASP 24 h *n* = 11, other CASP groups *n* = 12)

The activities of AST, ALT, and LDH in plasma were assessed as markers of liver injury. Plasma AST was elevated 24 h after sham and CASP surgeries and 48 h after the CASP operation compared to healthy controls. In contrast, plasma ALT and LDH were not elevated after any operation at any time point studied (Table 2).

### Effect of sterile laparotomy and abdominal infection on hepatic mitochondrial function

**Fig. 3:**
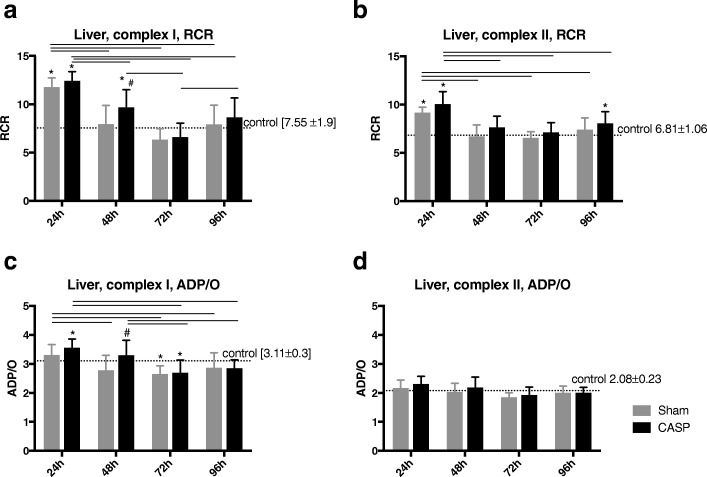
The RCR (state 3/state 2) for complex I (**a**) and complex II (**b**) was measured in liver homogenates from each group (control, straight horizontal dotted line; sham-operated, light grey bars; CASP-operated, dark grey bars), in a time-dependent manner. The ADP/O ratio for complex I (**c**) and complex II (**d**) in liver homogenates of the same groups was also measured. *p < 0.05 vs. control, #*p* < 0.05 vs. sham, between groups, data are shown as mean ± SD (control *n* = 9, sham *n* = 12, CASP *n* = 11–12, the value for each animal was calculated as the mean of three technical replicates)

Both the respiratory control ratio (RCR) and ADP/O were measured to assess the effects of sterile laparotomy and abdominal infection on mitochondrial function. In both sham-operated and CASP animals, the RCR for both complexes was elevated at 24 h after surgery compared to control animals. At all other defined time points, the RCR for both complexes in sham-operated animals was not significantly different from that in healthy controls (complex I Fig. 3a; complex II Fig. 3b). After CASP surgery, however, the RCR for complex I was also elevated at 48 h and for complex II at 96 h compared to unoperated controls (Fig. 3a, b).

The values of state 2 and state 3 are presented as Additional file 1: Table S1.

ADP/O ratio for complex I declined in sham animals, similar to CASP-operated animals, after 72 h (Fig. 3c), but remained unchanged for complex II over the course of time (Fig. 3d).

In CASP groups, the ADP/O ratio for complex I was elevated after 24 h (vs. healthy controls) and after 48 h (vs. sham-operated animals) and declined after 72 h (vs. healthy controls) (Fig. 3c). The ADP/O ratio for complex II stayed unchanged over the time period until 96 h (Fig. 3d).

### Effect of sterile laparotomy and abdominal infection on mitochondrial function in the colon

While hepatic mitochondrial function varied between sham- and CASP-operated animals, and over the entire time period, no changes were observed in colonic mitochondrial function between the groups, and only minor differences were seen among CASP-operated animals over time. However, the ADP/O ratio in the CASP group for complex II was significantly higher at 48 and 72 h compared to 24 h (Table 3).

**Table 3:** Respiratory control ratio (state 3/state 2) for complexes I and II and ADP/O ratio for complexes I and II in the colon homogenate, data are shown as mean ± SD (control *n* = 9, sham *n* = 12, CASP *n* = 11–12, the value for each animal was calculated as the mean of three technical replicates)

	Colon, RCR, complex I	Colon, RCR, complex II	Colon, ADP/O, complex I	Colon, ADP/O, complex II
Control	5.60 ± 1.73	5.05 ± 1.02	1.43 ± 0.53	0.99 ± 0.17
Sham 24 h	4.46 ± 1.21	4.33 ± 0.96	1.59 ± 1.61	0.91 ± 0.64
CASP 24 h	5.60 ± 1.67	4.99 ± 0.73	1.46 ± 1.40	0.72 ± 0.20
Sham 48 h	5.24 ± 2.23	4.70 ± 0.66	1.56 ± 0.40	1.15 ± 0.24
CASP 48 h	4.86 ± 1.49	5.34 ± 2.12	1.96 ± 0.64	1.38 ± 0.57
Sham 72 h	5.32 ± 1.66	4.87 ± 1.01	1.95 ± 0.68	1.22 ± 0.50
CASP 72 h	5.16 ± 1.50	5.10 ± 0.98	1.98 ± 0.69	1.34 ± 0.50
Sham 96 h	5.23 ± 1.98	4.64 ± 0.87	1.94 ± 0.71	1.09 ± 0.40
CASP 96 h	5.14 ± 2.27	4.77 ± 1.41	2.34 ± 1.74	1.20 ± 0.20

### Effect of sterile laparotomy and abdominal infection on the levels of lipid peroxidation in the liver and colon

**Fig. 4:**
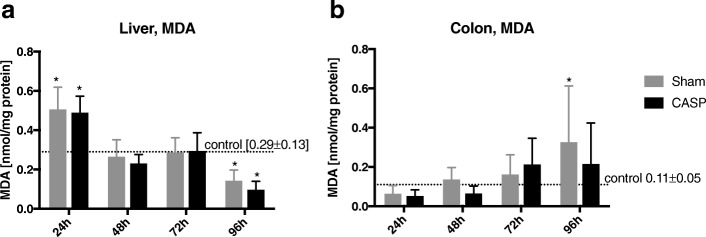
Assessment of MDA levels in the liver and colon tissue homogenates. MDA levels were detected by condensation with thiobarbituric acid and normalized to protein concentration in the liver (**a**) and colon (**b**) tissue homogenates. Data are shown as mean ± SD, *p < 0.05 vs. healthy controls,  between groups (control *n* = 9, sham *n* = 12, CASP *n* = 11–12)

Lipid peroxidation products, as markers of oxidative stress, were determined by detection of malondialdehyde (MDA). In the liver, the concentration of MDA was significantly higher in both operated groups after 24 h compared to healthy controls. The level of MDA continued to decline progressively to the level of healthy controls after 48 and 72 h and further decreased significantly after 96 h (Fig. 4a). In contrast, MDA levels in the colon in operated animals were not changed compared to unoperated controls. Compared to the control group, only a moderate increase was observed in sham-operated animals after 96 h (Fig. 4b).

## Discussion

The aim of the present study was to analyse time-dependent changes of mitochondrial respiration induced by abdominal infection in hepatic and colonic tissues. The main results show that coupling between respiratory chain and oxidative phosphorylation in the liver was higher in the early phase after the CASP operation (until 48 h). The efficacy of the oxidative phosphorylation was also higher up until 48 h, but lower after 72 h and normalized within the longer time period (until 96 h) after the CASP operation. In the first postoperative phase up until 24 h, the hepatic mitochondrial respiration was similarly modulated after sterile laparotomy and abdominal infection. Early changes of mitochondrial function (after 24 h) were observed for complexes I and II, whereas the later changes occurred only after stimulation of the respiratory chain through complex I. These results are in line with Brealey et al. who showed that the activities of the individual respiratory chain complexes changed differentially over time [6]. In contrast, colonic mitochondrial respiration was largely unaffected after both interventions.

The chosen CASP procedure represents a well-evaluated animal sepsis model because it mimics the abdominal infection in humans, such as that induced by anastomotic failure [27]. The assessment of organ damage markers (AST, ALT, LDH, creatinine, and urea) revealed only slight increases in the early postoperative phase with complete recovery within the time period over 96 h, confirming mild infection severity in our experimental model. Moderate infection was intended in our experimental setting to allow the investigation of later time points of up to 96 h without the risk of animal death. Animals did not receive antibiotics or additional fluid therapy, so the course of the abdominal infection was not modulated. All animals received analgesia over the whole observation time period (see the “Materials and methods” section). If buprenorphine did have any effect, this should be similar in all animals.

We measured mitochondrial respiration in tissue homogenates rather than in isolated intact mitochondria in order to avoid the further strain of mechanical isolation [28, 29]. To exclude mitochondrial damage due to the preparation procedure, we verified the integrity of the mitochondrial inner and outer membranes (see paragraph in section “Measurement of mitochondrial respiratory rates” in the section “Materials and methods”). Thus, the observed changes in mitochondrial function were caused by operative stress and/or abdominal infection and not by potential mechanical damage.

Hence, the experimental model chosen was appropriate to study the time-dependent effects of laparotomy and abdominal infection on mitochondrial function in the liver and colon.

The clinical and biological/immunological (IL-6, IL-10, TNF-α) signs of inflammation were more pronounced in septic rats compared to sham-operated rats.

Levels of pro- and anti-inflammatory cytokines were increased predominantly in the CASP groups and in the early (until 48 h) postoperative phase. TNF-α levels showed another peak after 96 h in both operated groups. The observation of higher levels of pro- and anti-inflammatory cytokines in the early phase of abdominal infection (until 48 h) is in line with the results of Osuchowski et al. They observed that the cytokine-based inflammatory response in the early phase of murine sepsis exhibits features of the mixed anti-inflammatory response syndrome (MARS) rather than SIRS [30]. Also, the later peaks of proinflammatory cytokines in chronic sepsis have been described [31]. Osuchowski et al. showed spikes of proinflammatory biomarkers in plasma at the late phase of sepsis. However, in contrast to this study, they used a lethal model of sepsis [31]. In addition, the levels of pro- and anti-inflammatory cytokines varied widely and were rather correlated with severity of sepsis than with the observation time points [30–32]. Our model was mild with a very low mortality rate, so it is still not clear why TNF-α had risen after 96 h and in both operated groups.

In a previous study, we observed that the severity of sepsis modulates mitochondrial function in the liver. Operative stress induced by laparotomy and, to a greater extent, moderate sepsis led to higher energy coupling, without affecting the efficacy of oxidative phosphorylation, whereas more severe sepsis did not affect hepatic mitochondrial function [18].

In this study, we focused on time-dependent and organ-specific effects and investigated mitochondrial respiration in the liver and colon over 96 h after laparotomy or CASP surgery. Mitochondrial respiration in the liver varied time-dependently, and the observed changes early after laparotomy and the CASP operation were similar. In the early postoperative phase (24 h), both sterile laparotomy and moderate sepsis elevated oxidation and phosphorylation (OxPhos) after stimulation through both complexes. In the further course, after 48 h, only abdominal infection increased the level of OxPhos for complex I. These results suggest that abdominal infection affects the individual complexes of the respiratory chain in different ways. This is in line with a study of Brealey et al. who could demonstrate that complex activities changed differently in the time period of 72 h after intraperitoneal injection of faecal slurry [14].

The efficacy of oxidative phosphorylation was modulated only for complex I. In the early postoperative phase, until 48 h, abdominal infection increased the efficacy of oxidative phosphorylation. In the later phase (72 h), sterile laparotomy and abdominal infection attenuated this response.

These results indicate that during the initial postoperative phase, i.e. from 24 to 48 h after sterile laparotomy or the CASP operation, OxPhos increased, then decreased in the later postoperative phase (72 h) and returned back to the levels of the healthy controls after 96 h. These results provide evidence that hepatic mitochondria adapt to a potentially minatorial state like inflammation [24].

Another possible explanation for these changes could be the varying cellular demand of ATP. Mitochondria are able to adjust their ATP production to the cellular requirements of energy [33, 34]. We did not assess the ATP demand in tissues, so we cannot fully exclude the possibility that the time-dependent changes in efficacy of the oxidative phosphorylation are partially caused by cellular ATP demand.

Even though the colon was the focus of inflammatory reaction, we did not observe any changes in the mitochondrial function in the colon tissue compared to healthy controls or to sham animals in the time period of 96 h after sham or CASP surgery. To our knowledge, there is no data about colonic mitochondrial function during sepsis in the literature. Mittal et al. have shown mitochondrial dysfunction of complex I and II pathways in the jejunum 6 h after pancreatitis induction [21]. However, the comparison with this study is highly limited because of the use of a different sepsis model, different time points, and the different tissue investigated. It is known that in the colonic section of the gut, a steep oxygen gradient exists, extending from the anaerobic lumen across the epithelium to the richly vascularized subepithelial mucosa. To maintain intestinal homeostasis, the colonic cells developed many mechanisms to cope with their austere metabolic environment [35]. One of these mechanisms is the stabilization of hypoxia-inducible factor (HIF), which enables epithelial cells to function effectively as a barrier under hypoxic conditions [36]. Hypoxia and inflammation share an interdependent relationship; on the one side, hypoxia elicits tissue inflammation, and on the other side, during inflammation, the inflammatory organ becomes ischemic [37]. This could, at least partially, explain why intestinal mitochondria do not change their function under inflammatory conditions with a substantial shift in metabolism and O_2_ availability. Moreover, small changes in O_2_ consumption observed in the colon might also be caused by a lower demand of ATP in this tissue [38].

Similar to the mitochondrial function, the ROS production in the liver was time-dependently modulated by sterile laparotomy and abdominal infection in a similar manner. The oxidative stress reactions in the liver, measured as lipid peroxidation in terms of malondialdehyde (MDA) equivalents, were significantly higher in both operated groups in the early postoperative phase (after 24 h), then declined (after 48 and 72 h) to the level of healthy controls and decreased significantly further after 96 h. As mitochondria are the main source of oxygen free radicals inducing oxidative stress in the cell, it is conceivable that enhanced mitochondrial activity in the early postoperative phase leads to increased ROS production [9, 39], which facilitate induction of oxidative stress. Our results are in line with Victor et al. who showed that ROS production in the liver was markedly increased in the early phase after induction of sepsis (6–18 h), followed by a subsequent decrease [40].

ROS production in colonic tissue followed a different pattern than in the liver. The largely unaffected MDA levels in operated animals compared to healthy controls were consistent with the unaltered mitochondrial function in our experiments. While a slight, continuous, time-dependent increase in the ROS production within both operated groups without a difference between the groups was observed, only the sham group reached the statistical significance over the controls after 96 h. This increase could possibly be explained by the elevated TNF-α level after 96 h, since TNF-α is known to accelerate ROS production [41]. However, TNF-α does not seem to be the sole factor influencing ROS levels since significant changes occurred only in sham animals but not in CASP animals and only in the colon. On the contrary, ROS production in the liver was significantly decreased after 96 h after sham and CASP surgery due to an unclear mechanism that requires further investigation. In experimental models of inflammatory bowel disease, the authors observed elevated ROS production in the colon [42]. They used trinitrobenzene sulphonic acid (TNBS)-induced colitis in rats, which probably leads to more pronounced inflammatory damage in colonic tissue than CASP and, therefore, possibly to increased ROS production.

### Limitations of the study

This study has certain limitations. The chosen animal model is of mild severity with a low mortality rate and without relevant signs of organ dysfunction. In this study, moderate infection was intended to allow the investigation of later time points of up to 96 h without a high rate of animal loss, but it did not allow us to compare the clinical phenotype with changes in mitochondrial respiration.

## Conclusions

Taken together, our results suggest that an abdominal infection modulates mitochondrial respiration in the liver in a time-dependent manner and, in the first 24 h, in a way similar to sterile laparotomy. In the early postoperative phase (until 48 h), when signs of inflammation are present, mitochondrial respiratory performance is increased. After 72 h, the mitochondrial respiration appears reduced (state 3 deteriorated) especially for complex I (lowered ADP/O ratio). In the late postoperative phase, after 96 h, mitochondrial respiration recovers.

Mitochondrial respiration in the colon seems to stay largely unaffected by abdominal infection. This is surprising because the colon is a focus of the inflammatory reaction in our model and also an organ which is more affected by microcirculatory dysfunction and, therefore, by impaired oxygen supply during sepsis. The possible explanation could be that the colonic cells are better adapted to varying oxygen concentrations and/or have, in general, a rather low metabolic activity [35, 38].

Although the transfer of the results obtained in rodents to human patients must be performed very carefully, the clinical impact could be that under inflammatory conditions, oxygen utilization is more effective. Even if oxygen supply is impaired, the cell metabolism is preserved. However, the exact explanation of this phenomenon needs further examination.

## Additional file

Additional file 1: **Table S1.** state 2 and state 3 for liver mitochondria stimulated through complexes I and II. Data are shown as mean ± SD, *p < 0.05 vs. control (controls *n* = 9, sham *n* = 12, CASP 24 h *n* = 11, other CASP groups *n* = 12), the value pro animal was calculated as a mean of three technical replicates. (DOCX 16 kb)

## Abbreviations

ADP/O ratio: Adenosine diphosphate-oxygen ratio
ALT: Alanine aminotransferase
AST: Aspartate aminotransferase
ATP: Adenosine triphosphate
BSA: Bovine serum albumin
CASP: Colon ascendens stent peritonitis
EDTA: Ethylenediaminetetraacetic acid
G-M: Glutamate-malate
HIF: Hypoxia-inducible factor
IL-10: Interleukin 10
IL-6: Interleukin 6
LDH: Lactate dehydrogenase
MARS: Mixed anti-inflammatory response syndrome
MDA: Malondialdehyde
MODS: Multiorgan dysfunction syndrome
MOPS: Morpholinepropanesulfonic acid
OxPhos: Oxidation and phosphorylation
RCR: Respiratory control ratio
ROS: Reactive oxygen species
S: Succinate
SIRS: Systemic inflammatory response syndrome
TBA: 2-Thiobarbituric acid
TNBS: Trinitrobenzene sulphonic acid
TNF-α: Tumour necrosis factor-α
