# Supplementary material for: Time-related changes in hepatic and colonic mitochondrial oxygen consumption after abdominal infection in rats
Source: Intensive Care Med Exp. 2019 Jan 8;7:4. doi: 10.1186/s40635-018-0219-9 (PMC6325055; doi:10.1186/s40635-018-0219-9)
Supplement: Supplementary file 1 — Table S1. state 2 and state 3 for liver mitochondria stimulated through complexes I and II. Data are shown as mean ± SD, *p < 0.05 vs. control (controls n = 9, sham n = 12, CASP 24 h n = 11, other CASP groups n = 12), the value pro animal was calculated as a mean of three technical replicates. (DOCX 16 kb) [file 40635_2018_219_MOESM1_ESM.docx]

|  | Liver, state 2, complex I  [nmol/min/mg] | Liver, state 2, complex II  [nmol/min/mg] | Liver, state 3, complex I  [nmol/min/mg] | Liver, state 3, complex II  [nmol/min/mg] |
| --- | --- | --- | --- | --- |
| Control | 1.79±0.31 | 2.73±0.38 | 13.13±2.78 | 18.64±4.22 |
| Sham 24 h | 1.14±0.11* | 2.12±0.23 | 13.38±1.13 | 19.33±1.89 |
| CASP 24 h | 1.19±0.17* | 2.13±0.26 | 14.68±1.96 | 20.69±2.70 |
| Sham 48 h | 1.43±0.29* | 2.33±0.32 | 11.20±2.52 | 15.40±2.95 |
| CASP 48 h | 1.38±0.35* | 2.42±0.63 | 12.85±2.88 | 18.14±4.34 |
| Sham 72 h | 1.49±0.31 | 2.17±0.54 | 9.22±1.93* | 13.96±3.14* |
| CASP 72 h | 1.44±0.30* | 2.00±0.42 | 9.30±2.23* | 14.21±3.73* |
| Sham 96 h | 1.55±0.46 | 2.39±0.64 | 11.96±3.25 | 17.43±4.56 |
| CASP 96 h | 1.41±0.49* | 2.20±0.61 | 11.54±3.36 | 17.53±5.18 |

Table S1. State 2 and State 3 for liver mitochondria stimulated through complex I and II. Data are shown as mean±SD, * p<0.05 vs. control (controls n=9, sham n=12, CASP 24h n=11, other CASP groups n=12), the value pro animal was calculated as a mean of three technical replicates).
